# Inhibition of 11β-hydroxysteroid dehydrogenase 1 alleviates pulmonary fibrosis through inhibition of endothelial-to-mesenchymal transition and M2 macrophage polarization by upregulating heme oxygenase-1

**DOI:** 10.1038/s41419-025-07522-2

**Published:** 2025-03-21

**Authors:** Su-Yeon Lee, Ji-Hee Kim, Yeonhwa Song, Sanghwa Kim, Hyo Jin Kang, Jason Kim, Yoon-Jin Lee, Haeng Ran Seo

**Affiliations:** 1 Advanced Biomedical Research Lab, Institut Pasteur Korea, 16, Daewangpangyo-ro 712 beon-gil, Bundang-gu, Seongnam-si, Gyeonggi-do 13488 Republic of Korea; 2 Division of Radiation Effects, Korea Institute of Radiological and Medical Sciences, 75 nowongu nowon gil, Seoul, 139-706 Korea; 3 R&D center, J2H Biotech Inc., Saneop-ro 156 beon-gil, Gwonseon-gu, Suwon-si, Gyeonggi-do 16648 Republic of Korea

**Keywords:** Diseases, Drug development

## Abstract

The intracellular enzyme 11β-hydroxysteroid dehydrogenase type 1 (11βHSD1) catalyzes the interconversion of active glucocorticoid (cortisol) and its intrinsically inert form (cortisone) in metabolic tissues. Although 11βHSD1 is considered a promising therapeutic target in metabolic disorders such as type 2 diabetes, obesity, and nonalcoholic steatohepatitis because of its hepatic functions, its roles in other tissues have received less attention. In this study, we show that the 11βHSD1-specific inhibitor J2H-1702 facilitates the reversion of endothelial-to-mesenchymal transition in multicellular lung spheroid models encapsulating the complex crosstalk among lung cancer cells, vascular endothelial cells, and macrophages. In vascular endothelial cells, J2H-1702 not only suppressed interleukin-1α (IL-1α) expression but also attenuated reactive oxygen species-induced DNA damage by upregulating heme oxygenase-1. Additionally, in macrophages, which are key regulators of fibrogenesis, inhibition of 11βHSD1 markedly reduced IL-1β expression, thereby modulating the pro-inflammatory phenotype of activated macrophages. In mouse models of pulmonary fibrosis, including a bleomycin-induced idiopathic model and a radiation-induced model, J2H-1702 alleviated pulmonary fibrosis and markedly improved the efficacy of nintedanib. Collectively, our data suggest that J2H-1702 holds promise as a clinical candidate for the treatment of pulmonary fibrosis associated with reactive oxygen species-induced DNA damage, endothelial-to-mesenchymal transition, and inflammatory responses.

## Introduction

Pulmonary fibrosis (PF) is a serious and progressive lung disease that occurs when lung tissue becomes damaged and scarred. The prognosis is poor, with a reported median survival time of 3.8 years [1]. Mechanisms of PF pathological development are not clearly understood because PF can have different causes or triggers; however, aberrant epithelial wound healing in association with dysregulated fibroblast proliferation and matrix synthesis is generally recognized to have a key role in PF development [2]. Pirfenidone and nintedanib are anti-fibrotic medications approved for PF treatment. Although both drugs slow PF progression [3, 4] and increase survival time [5, 6], they only partially delay the disease [7] by supporting lung function. There is currently no cure for PF.

The endothelial-to-mesenchymal transition (EndMT) is characterized by the loss of endothelial marker expression and acquisition of mesenchymal or fibroblastic phenotypes, including the production of smooth muscle actin (SMA), fibroblast-specific protein 1, and type I collagen, resulting in cells that have invasive and migratory potential [8]. The modulation of EndMT was recently proposed as a potential therapeutic strategy for various fibrotic diseases [9, 10]. EndMT has also been reported to play a critical role in pulmonary hypertension by causing the accumulation of mesenchymal-like cells in obstructive pulmonary vascular lesions [11]. Furthermore, cancer cells stimulate EndMT in endothelial cells (ECs) and transformed ECs support cancer progression by secreting cytokines, growth factors, and proteins of the endoplasmic reticulum membrane-protein complex.

Multicellular spheroid culture systems are believed to mimic three-dimensional environments in the body [12]. We previously showed that realistic reciprocal crosstalk between lung cancer cells and stromal cells within multicellular lung spheroids (MCLSs) causes those cells to recapitulate morphological changes that occur in vascular ECs during EndMT [13, 14]. To identify compounds that inhibit EndMT, we employed a visual phenomic screening platform that uses image-mining tools to screen cellular disease models and cellular phenotypes for small molecules that change observed phenotypes [15, 16]. In anti-fibrotic drug development, these systems can be combined with phenotypic screening to provide an efficient and high-throughput platform to identify new drugs and drug targets.

The enzyme 11β-hydroxysteroid dehydrogenase type 1 (11βHSD1) catalyzes the intracellular conversion of cortisone to physiologically active cortisol [17]. In a previous study, we found that J2H-1702, a novel 11βHSD1 inhibitor, ameliorated liver fibrosis by inhibiting the expression of pro-fibrotic genes [18]. A subsequent phase 1 study in healthy Korean males demonstrated that J2H-1702 not only was safe and well-tolerated but also had the ability to inhibit 11βHSD1 activity, with single ascending dose and multiple ascending dose studies showing that dose-dependent exposure in plasma was compatible with once-daily dosing within a therapeutic range [19]. Drug repurposing is an approach that identifies new clinical applications for approved or investigational drugs outside the drugs’ originally intended uses [20]. Because several pathways are concurrently activated or downregulated in hepatic fibrosis and pulmonary fibrosis [21], we investigated whether J2H-1702 might be effectively repurposed to treat PF.

In recent studies, growth factors family, which include TGF-βs, PDGFs, FGFs, and connective tissue growth factor (CTGF) [22], and antioxidants have been suggested as targets in hepatic fibrosis and pulmonary fibrosis. Similar to liver and lung fibrosis, the induction of the TGF-β/SMAD pathway by antioxidants is also seen in renal fibrosis [23]. Among the antioxidants, heme oxygenase-1 (HO-1) has been highlighted in several fibrosis [24, 25].

Specifically, we asked whether J2H-1702 can reverse fibrotic properties and inhibit EndMT-promoting signals in MCLSs. In addition, we searched for a mechanism by which J2H-1702 could inhibit PF. Our findings suggest that J2H-1702 has the potential to be repurposed as a novel treatment for PF.

## Results

### The 11βHSD1 inhibitor J2H-1702 reduces EndMT in MCLSs

**Fig. 1:**
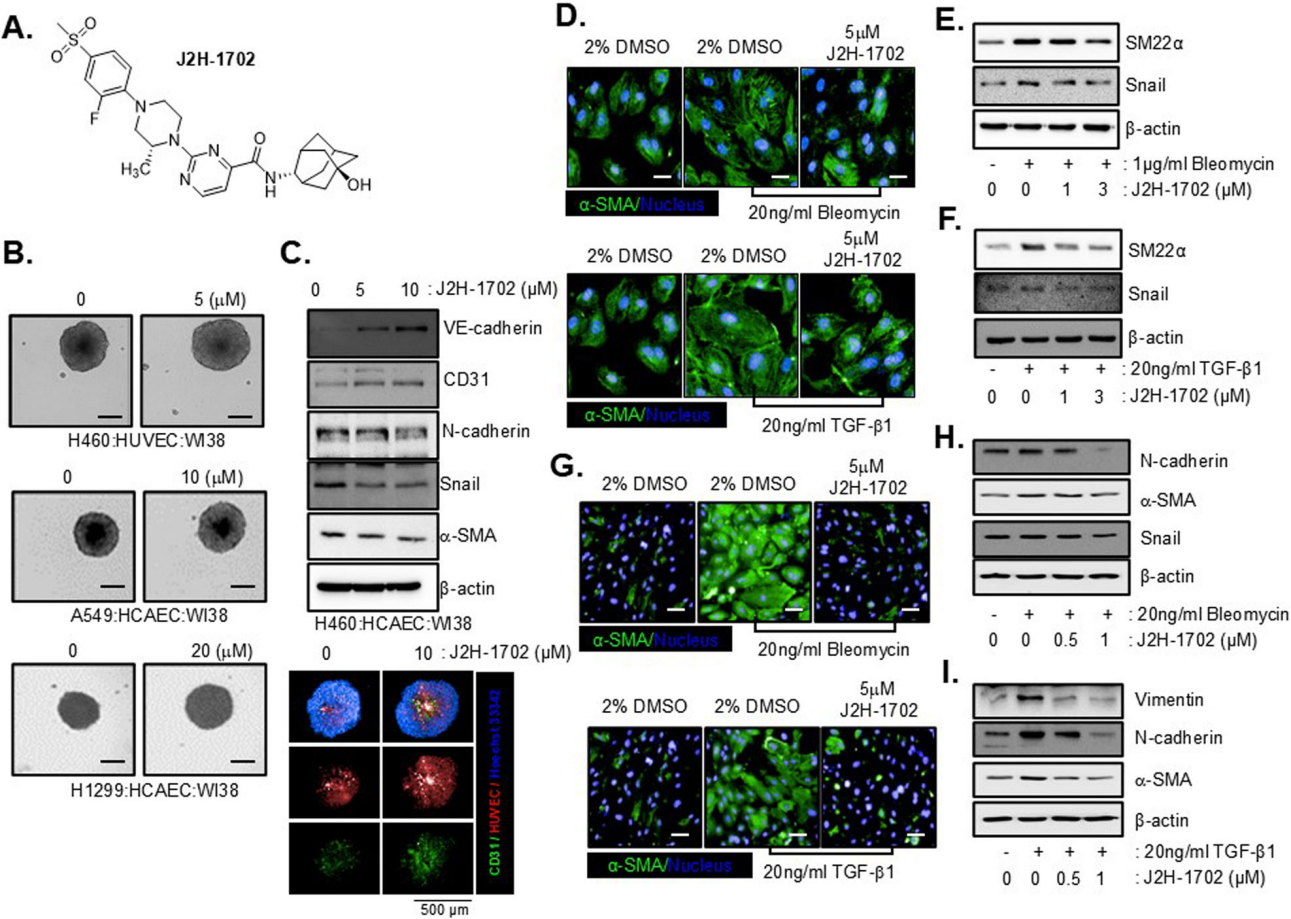
The 11βHSD1 inhibitor J2H-1702 inhibits endothelial-to-mesenchymal transition in multicellular lung spheroids. **A** The chemical structure of J2H-1702. **B** Various types of multicellular lung spheroids (MCLSs; H460:HUVEC:WI38, H460:HCAEC:WI38, H1299:HCAEC:WI38) were formed and treated with the indicated concentrations of J2H-1702 for 3 days. **C** H460:HCAEC:WI38 MCLSs were treated with J2H-1702 for 3 days. Cells were then harvested and lysed. The expression of endothelial cell markers (VE-cadherin, CD31) was increased in a dose-dependent manner, and the expression of mesenchymal markers (N-cadherin, Snail, α-SMA) was decreased (upper). J2H-1702 treated H460 MCLSs were stained with CD31 (green), among them, HUVEC cells were stained with DiD lipophilic tracer (red), and nuclei were stained with Hoechst 33342 (blue) (under). **D** Human coronary artery endothelial cells (HCAECs) were stained for α-SMA (green) and nucleus (blue). **E**, **F** Bleomycin-induced (**E**) or TGF-β1–induced (**F**) HCAECs were lysed, and a western blot was performed. Protein expression of SM22α and Snail was reduced by treatment with J2H-1702. **G** HUVECs were stained for α-SMA (green) and nucleus (blue). **H**, **I** Bleomycin-induced (**H**) or TGF-β1–induced (**I**) HUVECs were lysed, and a western blot was performed. The protein expression of endothelial-to-mesenchymal transition markers was reduced by treatment with J2H-1702. Scale Bar: 20 μm. **B**, **C** Experiments were performed in MCLSs (3D spheroid). **D**–**I** All experiments were performed in 2D monolayer.

The structure of J2H-1702 is shown in [Fig. 1A]. In a previous study, we proposed that J2H-1702 can ameliorate liver fibrosis by inhibiting pro-fibrotic gene expression and TGF-β1-mediated collagen synthesis, α-SMA production, and Smad2/3 phosphorylation in a fibrotic environment. Here, because these mechanisms would exert inhibitory effects on EndMT as well as epithelial-to-mesenchymal transition before the development of tissue fibrosis, we decided to further investigate the effect of J2H-1702 on EndMT in MCLSs. To model the complexity and heterogeneity of lung fibrosis in vitro, we generated MCLS models with various combinations of lung cancer cell lines, lung fibroblasts, and ECs. EndMT is an important source of fibrotic cells in PF, so we used two types of ECs: human umbilical vein endothelial cells (HUVECs) and human coronary artery endothelial cells (HCAECs). When we investigated architectural changes in the MCLSs following J2H-1702 treatment, we observed prominent increases in the size of J2H–1702–treated MCLSs relative to that of DMSO-treated MCLSs [Fig. 1B]. To determine if J2H-1702 caused a difference in EndMT activity in the MCLSs, we measured the expression of CD31 and VE-Cadherin as EC markers and N-cadherin, Snail, and α-SMA as mesenchymal markers. The expression of CD31 and VE-Cadherin, which was suppressed in untreated MCLSs, was recovered by J2H-1702 treatment, whereas the expression of N-cadherin, Snail, and α-SMA, which was overexpressed in untreated MCLSs, was inhibited by J2H-1702 treatment. In addition, the result of immunofluorescent staining showed that the expression of CD31 in H460 MCLSs was increased by J2H-1702 treatment and also we observed that the size of the spheroid was bigger than untreated MCLSs. H1299 MCLSs have similar effects to H460 MCLSs. Data not shown [Fig. 1C].

We conducted cellular phenotype assays to confirm the effect of J2H-1702 on the EndMT activity of ECs. Bleomycin can induce inflammation and fibrosis by activating TGF-β1 signaling in the lungs. The proliferation of α-SMA fibers is a proper morphometric signature of EndMT. Treatment of HCAECs with 20 ng/ml bleomycin or 20 ng/ml TGF-β1 resulted in the accumulation of α-SMA fibers, which was attenuated by additional J2H-1702 treatment [Fig. 1D]. Furthermore, bleomycin treatment in HCAECs resulted in overexpression of the EndMT markers SM22α and Snail, which was inhibited by additional treatment with 3 μM J2H-1702 [Fig. 1E]. J2H-1702 treatment also reduced the expression of SM22α and Snail in TGF-β1-treated HCAECs [Fig. 1F]. When HUVECs were used in cellular phenotype assays instead of HCAECs, we detected similar effects of J2H-1702 against EndMT activity [Fig. 1G]. Similarly, the upregulated expression of mesenchymal markers induced by bleomycin [Fig. 1H] or TGF-β1 [Fig. 1I] was reduced by J2H-1702 treatment in HUVECs. These results suggest that the 11βHSD1 inhibitor J2H-1702 can suppress the EndMT process in ECs.

### J2H-1702 increases Heme oxygenase 1 expression in MCLSs undergoing EndMT

**Fig. 2:**
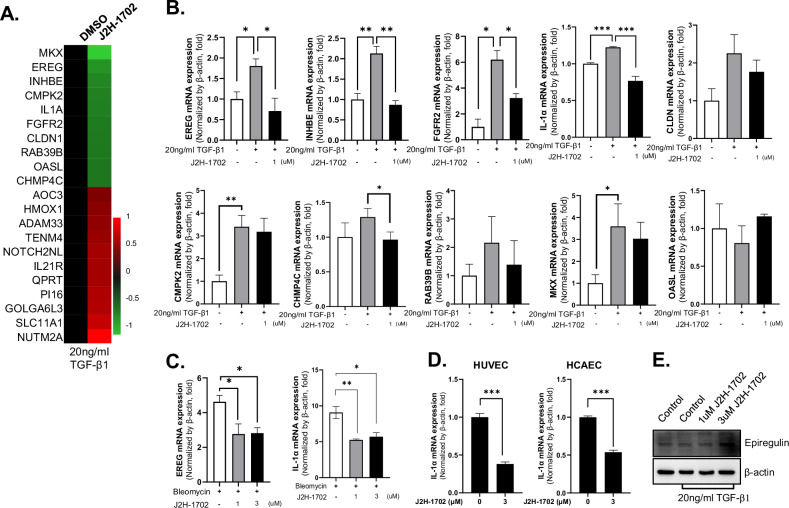
J2H-1702 controls endothelial-to-mesenchymal transition activity in HUVECs. **A** RNA sequencing analysis of HUVECs treated with 20 ng/ml TGF-β1 alone or in combination with J2H-1702. RNA sequencing analysis data are described in supplementary material. **B** mRNA expression of EREG, INHBE, FGFR2, IL-1α, CLDN, CMPK2, CHMP4C, RAB39B, MKX, and OASL was analyzed in HUVECs treated with 20 ng/ml TGF-β1 alone or in combination with 1 μM J2H-1702. **C** mRNA expression of EREG and IL-1α was analyzed in HUVECs treated with 10 μg/ml bleomycin alone or in combination with 1 or 3 μM J2H-1702. **D** IL-1α mRNA expression was measured in HUVECs treated with 3 μM J2H-1702. **E** Protein expression of Epiregulin in HUVECs was detected by western blot. Data are shown as the mean ± standard deviation (*n* = 3). *P < 0.05, **P < 0.01, and ***P < 0.001.

Because our phenotypic results showed that J2H-1702 could control EndMT activity, we used genome-wide RNA sequencing (RNA-seq) to investigate the mechanism by which J2H-1702 suppresses TGF-β1-induced EndMT activation in HUVECs. We identified differentially expressed genes based on a cutoff of >3-fold difference in expression between HUVECs treated with TGF-β1 alone and HUVECs treated with TGF-β1 and J2H-1702. Analysis of the differentially expressed genes revealed that J2H-1702 downregulated genes related to fibroblast growth factor receptors, PI3K/epiregulin, the IL-1 pathway, glycoprotein hormones, and pyroptosis [Fig. 2A]. We used RT-PCR to further analyze the expression of the differentially expressed genes identified by RNA-seq. We found that the mRNA expression of *EREG*, *INHBE*, *FGFR2*, and *IL-1α* in TGF-β1-treated HUVECs was markedly reduced by additional J2H-1702 treatment [Fig. 2B]. The mRNA expression of *EREG* and *IL-1α* in bleomycin-treated HUVECs was also suppressed by J2H-1702 [Fig. 2C]. In addition, treatment with J2H-1702 alone effectively attenuated the mRNA expression of *IL-1α* in HUVECs and HCAECs. [Fig. 2D]. Despite the changes in mRNA expression, the changes in EREG protein expression induced by J2H-1702 were weak in TGF-β1-treated HUVECs. [Fig. 2E].

**Fig. 3:**
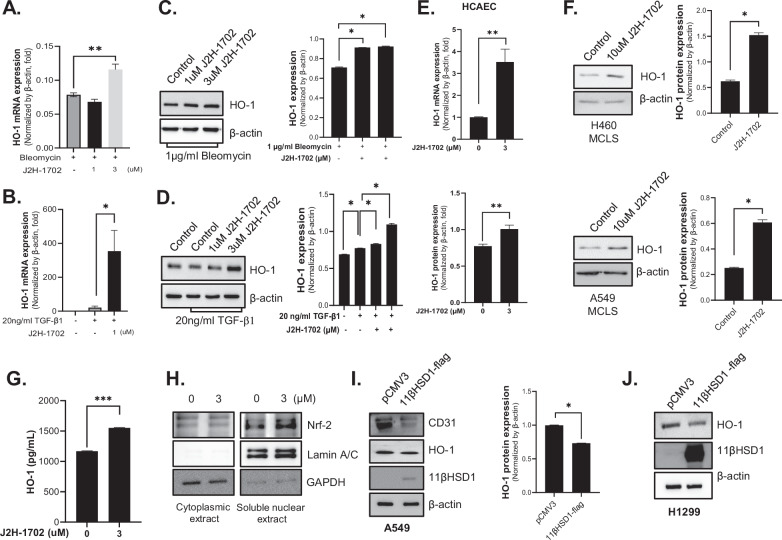
J2H-1702 increases Heme oxygenase-1 expression. Heme oxygenase-1 (HO-1) mRNA expression was increased by J2H-1702 in HUVECs treated with bleomycin (**A**) or TGF-β1 (**B**). **C**, **D** Protein expression of HO-1 in human coronary artery endothelial cells (HCAECs) was detected by western blot. The graph was analyzed using Image J software. **E** mRNA (upper) and protein (under) expressions of HO-1 following treatment with 3 μM J2H-1702 in HCAECs were increased. **F** Protein expression of HO-1 following treatment with 10 μM J2H-1702 in multicellular lung spheroids containing H460 cells (upper) and A549 cells (under) was detected by western blot. The graphs were analyzed using Image J software. **G** HO-1 ELISA of H1299 cells (2D cell) with or without administration of 3 μM J2H-1702. **H** HCAEC fractionation was performed, and expression of Nrf2 in the cytoplasmic and soluble nuclear extracts was detected. Lamin A/C was used as a nuclear marker, and GAPDH was used as a cytoplasmic marker. **I** A549 cells were transfected with pCMV3 empty vector or 11βHSD1-flag tagged plasmid, and western blot was performed. The protein expression of CD31 and HO-1 was decreased in 11βHSD1-overexpressing cells. The graph was analyzed using Image J software. **J** H1299 cells were transfected with pCMV3 empty vector or 11βHSD1-flag tagged plasmid, and western blot was performed. The protein expression of HO-1 was decreased in 11βHSD1-overexpressing cells. Data are shown as the mean ± standard deviation (*n* = 3). *P < 0.05, **P < 0.01, and ***P < 0.001. **A**–**E**, **G**–**J** Experiments were performed in 2D monolayer cells. Figure 3F, H460 MCLS, and A549 MCLS experiments were performed in 3D spheroid. Beta-actin in Fig. 3C is the same data as beta-actin in Fig. 1E. Western blot experiments of Figs. 1E and 3C were performed on the same day. The western blot bands were edited and displayed in Fig. 3C according to the flow of the manuscript.

Next, we focused on genes that were upregulated by J2H-1702 in TGF-β1–treated HUVECs. Among these genes, the mRNA expression of heme oxygenase 1 (HO-1), which plays a primary antioxidant and anti-inflammatory role in heme degradation, was increased in both bleomycin-treated HUVECs [Fig. 3A] and TGF-β1-treated HUVECs [Fig. 3B]. J2H-1702 also increased HO-1 protein expression in bleomycin-treated HCAECs [Fig. 3C] and TGF-β1-treated HCAECs [Fig. 3D]. Increased HO-1 expressions in both mRNA (upper) and protein (under) following J2H-1702 treatment were observed in HCAECs [Fig. 3E]. Protein expression of HO-1 following J2H-1702 treatment was also observed in MCLSs containing H460 cells (upper) and A549 cells (under) [Fig. 3F]. In addition to ECs, lung cells, which account for a large portion of MCLSs, also showed increased HO-1 protein levels after J2H-1702 treatment [Fig. 3G]. The Nrf2 transcription factor promotes heme oxygenase expression, and Nrf2/HO-1 signaling is a promising therapeutic target in PF [24]. J2H-1702 promoted Nrf2 nuclear translocation and induced HO-1 expression in HCAECs [Fig. 3H], whereas 11βHSD1 overexpression resulted in the inhibition of HO-1 expression in A549 cells [Fig. 3I] and H1299 cells [Fig. 3J].

### J2H-1702 attenuated reactive oxygen species-induced DNA damage in a fibrotic environment

**Fig. 4:**
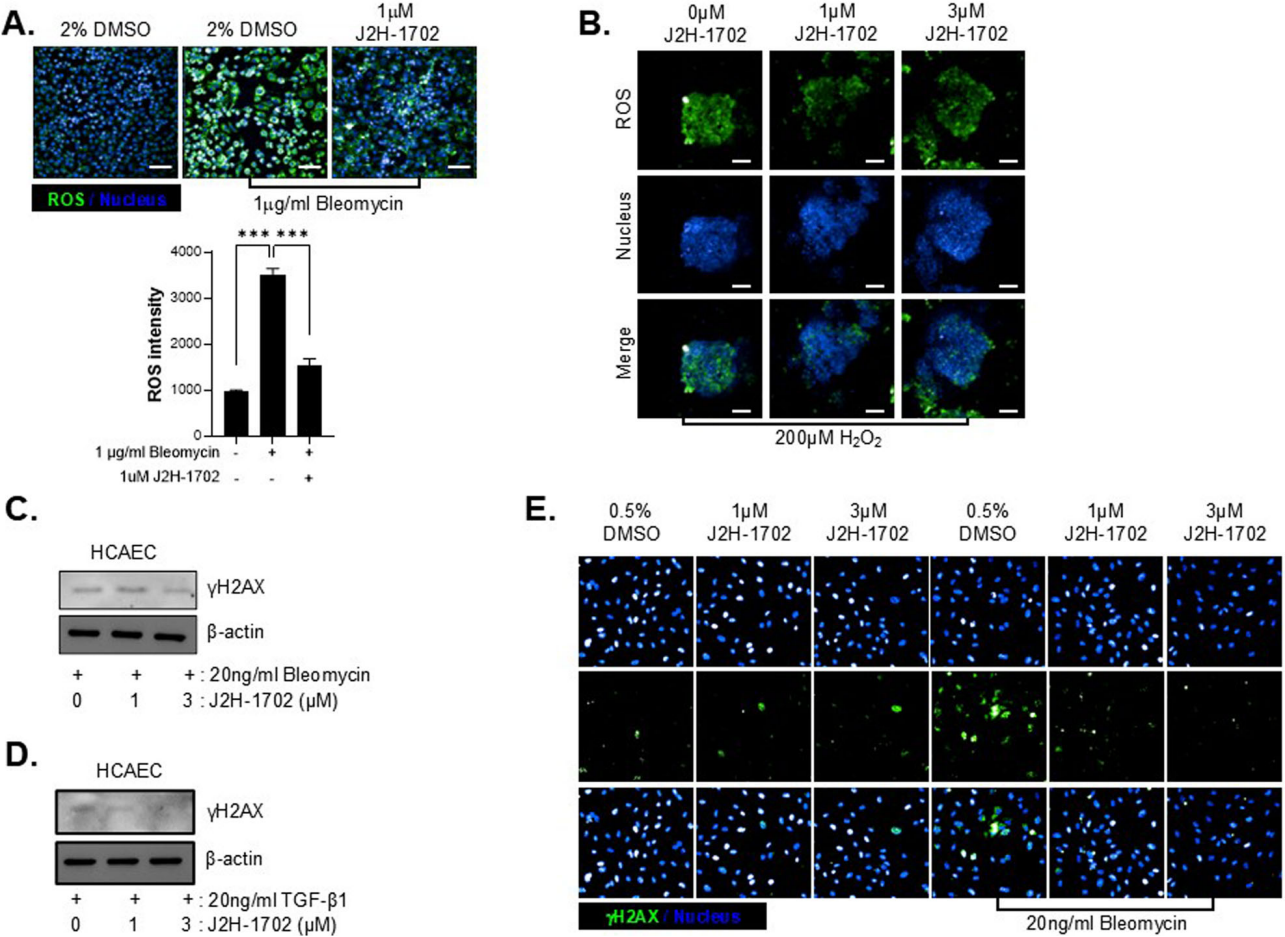
J2H-1702 attenuated reactive oxygen species-induced DNA damage in a fibrotic environment. **A** H1299 cells were treated with 1 μg/ml bleomycin alone or in combination with 1 μM J2H-1702. Live cells were stained with CM-H_2_DCFDA. Reactive oxygen species intensity was measured using Opera Phenix and analyzed using Harmony software. **B** H1299 cells in three-dimensional culture were incubated with 200 μM H_2_O_2_ for 1 h and then with 1 μM or 3 μM J2H-1702 for 48 h. CM-H_2_DCFDA staining was performed to detect reactive oxygen species, and Heochst 33342 staining was performed to detect nuclei. **C**, **D** Protein expression of γH2AX was measured in human coronary artery endothelial cells induced with bleomycin (**C**) or TGF-β1 (**D**). γH2AX expression was decreased in cells co-treated with J2H-1702. **E** HUVECs were seeded in a 384-well plate and stained with Hoechst 33342 and γH2AX antibody after fixation. The image was acquired using Opera Phenix. Data are shown as the mean ± standard deviation (*n* = 3). ***P < 0.001. Scale Bar: 20 μm.

Bleomycin-induced PF is associated with increases in reactive oxygen species (ROS) levels and DNA double-strand breaks. To determine whether the J2H-1702-induced increase in HO-1 expression affected cellular antioxidant capacity, we measured ROS accumulation in bleomycin-treated H1299 cells by fluorescence microscopy after staining with CM-H_2_DCFDA. The results showed that J2H-1702 dramatically reduced the bleomycin-induced ROS accumulation [Fig. 4A]. Next, we examined whether the J2H-1702-induced increase in HO-1 expression could modulate oxidative stress in MCLSs. We found that J2H-1702 treatment not only inhibited ROS accumulation following H_2_O_2_ exposure but also reduced the structural compactness of the MCLSs [Fig. 4B].

Because ROS are generally known as mediators of DNA damage, we next measured the effect of J2H-1702 on bleomycin-induced DNA damage. In bleomycin-treated HCAECs, J2H-1702 diminished the expression of γH2AX, which is a marker of DNA damage [Fig. 4C]. TGF-β1 accelerates the DNA damage response [26, 27], so we examined the effect of J2H-1702 on DNA damage caused by TGF-β1 treatment. We found that treatment with J2H-1702 reduced γH2AX expression in TGF-β1–treated HCAECs [Fig. 4D]. In HUVECs, treatment with 20 ng/ml bleomycin led to enough accumulation of γH2AX foci to fill the cell nucleus, and this accumulation was dramatically reduced by additional treatment with J2H-1702 [Fig. 4E]. These results suggest that J2H-1702 attenuates ROS-induced endothelial stress in a fibrotic environment.

### J2H-1702 controls macrophage polarization by inhibiting IL-1β

**Fig. 5:**
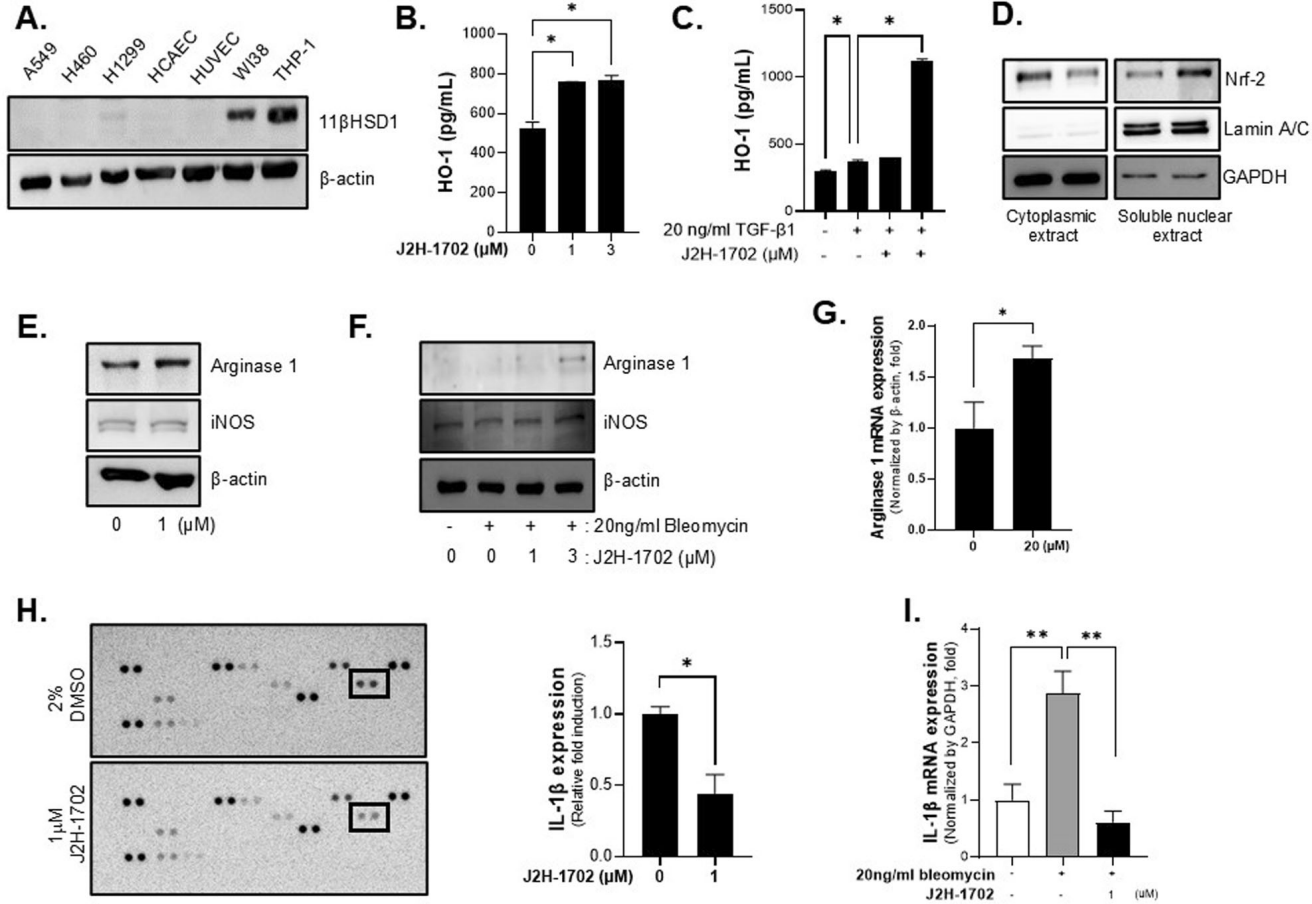
J2H-1702 controls macrophage polarization by inhibiting IL-1β. **A** Expression of 11βHSD1 in monolayer (two-dimensional) cultures of various cell lines (A549, H460, H1299, HCAEC, HUVEC, WI38, THP-1) was detected by western blot. **B**, **C** HO-1 ELISA was performed with THP-1 cells. HO-1 expression was increased by treatment with 1 μM or 3 μM J2H-1702 (**B**) or by co-treatment with J2H-1702 and TGF-β1 (**C**). **D** THP-1 cell fractionation was performed, and expression of Nrf2 in the cytoplasmic and soluble nuclear extracts was detected. Lamin A/C was used as a nuclear marker, and GAPDH was used as a cytoplasmic marker. **E**, **F** Western blot was performed with THP-1 cells. Expression of the M2 macrophage marker Arginase 1 was increased by J2H-1702 treatment (**E**) or by co-treatment with J2H-1702 and bleomycin (**F**). **G** Treatment with 20 μM J2H-1702 resulted in elevated Arginase 1 mRNA expression in immune-activated MCLSs containing H460 cells. **H** The proteome profiler human cytokine array was used to analyze THP-1 cells treated with 1 μM J2H-1702. The spot size was decreased for IL-1β, and the spot expression was normalized against reference spots. **I** mRNA expression of IL-1β was analyzed in bleomycin-induced THP-1 cells and treated with J2H-1702. Data are shown as the mean ± standard deviation (*n* = 3). *P < 0.05 and **P < 0.01.

We next investigated which cells within MCLSs predominantly expressed 11βHSD1. We found that lung cancer cells and HUVECs rarely expressed 11βHSD1, whereas THP-1 cells, which are widely used to study monocytes/macrophages, showed the highest 11βHSD1 expression among the cells used in the MCLSs [Fig. 5A]. When we measured HO-1 production in THP-1 cells, we found that J2H-1702 accelerated HO-1 production in THP-1 cells when it was applied alone [Fig. 5B] or in combination with TGF-β1 [Fig. 5C]. Cell fractionation results showed that similar to the results in HUVECs, J2H-1702 promoted Nrf2 nuclear translocation in THP-1 cells [Fig. 5D].

Because DNA damage affects the inflammatory microenvironment and ROS plays a critical role in macrophage polarization [28], we investigated the effects of J2H-1702 on macrophage polarization. Treatment of THP-1 cells with J2H-1702 upregulated the expression of Arginase 1, a marker of M2 macrophages, without altering the expression of iNOS, an M1 marker, both in the absence [Fig. 5E] and in the presence [Fig. 5F] of bleomycin co-treatment. We also observed increased expression of Arginase 1 in immune-activated MCLS models in which macrophages were co-cultured with the MCLSs [Fig. 5G].

Next, we screened the expression of human cytokines in J2H-1702-treated THP-1 cells. Among various cytokines, J2H-1702 selectively inhibited the pro-inflammatory cytokine IL-1β [Fig. 5H]. RT-PCR experiments confirmed that J2H-1702 effectively reduced IL-1β expression in bleomycin-treated THP-1 cells [Fig. 5I].

### J2H-1702 alleviates fibrosis in a bleomycin-induced PF model

**Fig. 6:**
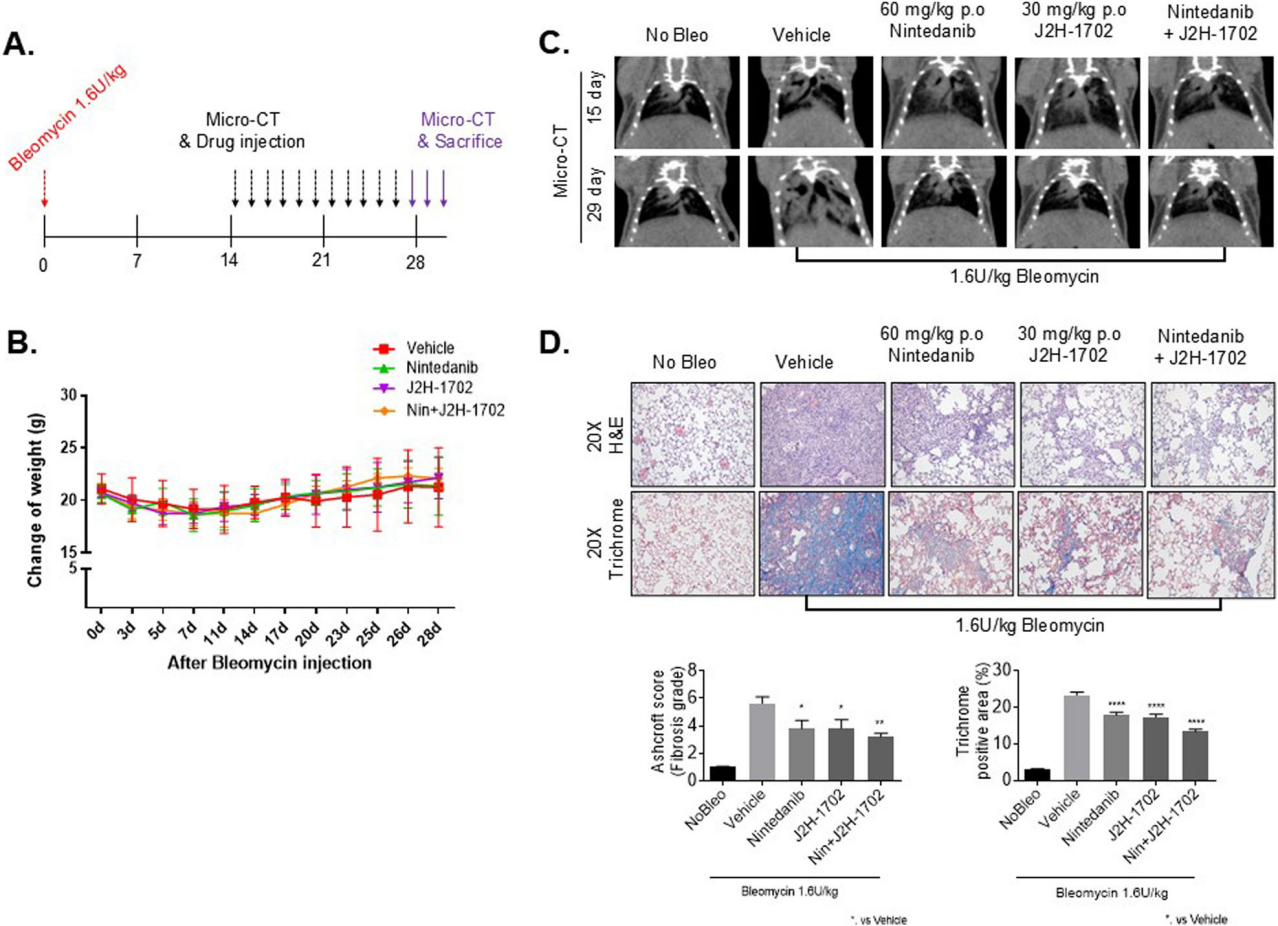
J2H-1702 improved fibrosis in a bleomycin-induced pulmonary fibrosis model. **A** Six-week-old male C57BL/6 N mice were administered bleomycin (1.6 U/kg) intratracheally once daily for 14 days. Drugs were then orally administered once daily for another 14 days. Micro-CT images were scanned during this period. **B** Body weight of the mice was measured for 28 days after the start of bleomycin treatment. **C** Micro-CT images were acquired on days 15 and 29. Gray areas appear as an accumulation of inflammatory consolidation. **D** H&E and Masson’s trichrome staining were performed in mice tissues. The fibrosis grade was evaluated according to the Ashcroft score, and collagen deposition was evaluated. (*n* = 7 animals per group). Error bars indicate the standard deviation (S.D); One-way ANOVA for multiple comparison. N.S., not significant. *P < 0.05, **P < 0.01, and ****P < 0.0001.

Among various animal models of PF, the bleomycin-induced model is the most widely used mouse model and has the best characteristics at present. To test the efficacy of J2H-1702 as a potential PF treatment, we administered 1.6 U/kg bleomycin intratracheally once daily to C57BL/6 mice for 14 days. Starting the day after the last bleomycin dose, the mice were orally administered 30 mg/kg J2H-1702 and/or 60 mg/kg nintedanib once daily for 14 days [Fig. 6A]. There was no drastic change in the body weight of the mice after drug administration [Fig. 6B].

Micro-CT imaging can visualize progressive anatomical changes in the lungs. The bleomycin-induced PT mice displayed gray areas in micro-CT scans, indicating accumulation of inflammatory exudate (consolidation) in the alveoli, which prevents air access. The bleomycin-induced PT mice also displayed pulmonary consolidation, which was reduced by further treatment with J2H-1702 or nintedanib. The combined administration of J2H-1702 and nintedanib had synergistic anti-fibrotic effects in the bleomycin-induced PF mice, substantially reducing the number of gray areas in micro-CT scans [Fig. 6C]. Analyses of Ashcroft scores and trichrome-positive areas in lung tissues from the bleomycin-induced PF mice showed that although treatment with either J2H-1702 or nintedanib significantly alleviated fibrosis, combined treatment with J2H-1702 and nintedanib had better efficacy than either treatment alone [Fig. 6D].

### J2H-1702 markedly improves the anti-fibrotic efficacy of nintedanib in a radiation-induced pulmonary fibrosis model

We employed a radiation-induced pulmonary fibrosis (RIPF) mouse model to validate the efficacy of J2H-1702 against lung fibrosis. Compared with normal tissues, irradiated lung tissues displayed morphologic changes in the irradiated area 2 weeks after mice were administered 90 Gy focal irradiation from a 4-mm collimator. The irradiated areas exhibited a definite, white, ring-like boundary with white-colored adjacent areas, indicating lung inflammation. RIPF mice were administered J2H-1702, nintedanib, or both J2H-1702 and nintedanib orally twice daily for 2 weeks. During the observation period, no remarkable changes, such as altered movement or fur shine, were observed, but diarrhea was observed in mice that received nintedanib. There was no drastic change in the body weight of the mice after drug administration, but the mice that received nintedanib showed a slight decrease in body weight 3 days after drug administration, followed by a gradual increase.

**Fig. 7:**
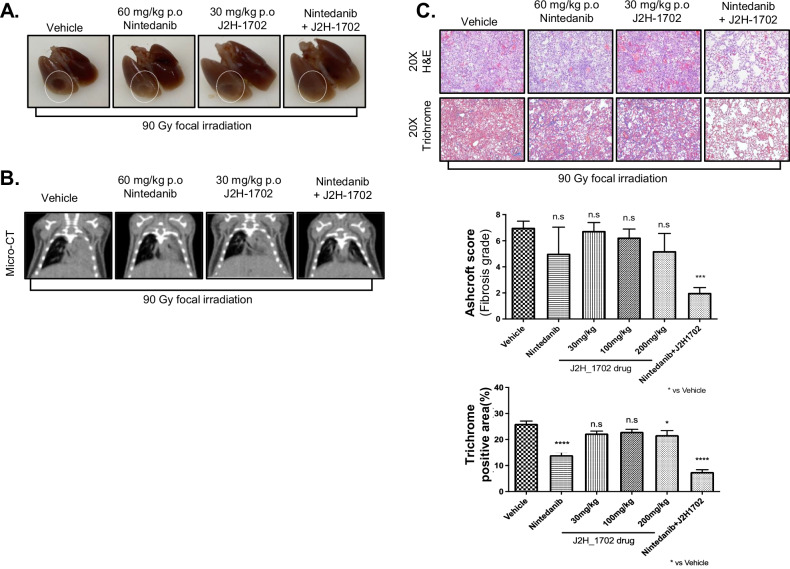
J2H-1702 reduces fibrosis in a radiation-induced pulmonary fibrosis mouse model. Male 7-to-8-week-old C57BL/6 mice were irradiated with 90 Gy using a 4 mm diameter field. **A** The radiation-induced white ring boundary in the lungs exhibits morphological changes according to drug administration. **B** Micro-CT images were acquired after drug administration. **C** H&E and Masson’s trichrome staining were performed in mice tissues. The fibrosis grade was evaluated according to the Ashcroft score, and collagen deposition was evaluated. (*n* = 4 animals per group). Error bars indicate the standard deviation (S.D); One-way ANOVA for multiple comparison. N.S, not significant. *P < 0.05, ***P < 0.001, and ****P < 0.0001.

Unlike the results of the bleomycin-induced PF model, treatment with either J2H-1702 or nintedanib had little effect on lung inflammation in the RIPF model. However, the combined treatment with J2H-1702 and nintedanib showed a clear anti-fibrotic effect [Fig. 7A]. The radiation-induced expansion of the extreme gray area on micro-CT images was effectively suppressed by the combined administration of J2H-1702 and nintedanib in the RIPF mice [Fig. 7B].

H&E and trichrome staining revealed that 90 Gy irradiation induced fibrotic changes and collagen deposition in the mouse lungs. An analysis of Ashcroft scores showed that although J2H-1702 had a dose-dependent effect in comparison with the vehicle, the effect was not highly significant; however, the combined J2H-1702 and nintedanib treatment showed the most significant effect on Ashcroft score among the treatment groups. Analyses of H&E and trichrome staining showed that all the drug treatments had anti-fibrotic effects compared with vehicle, but the nintedanib treatment and the combined J2H-1702 and nintedanib treatment had the strongest anti-fibrotic effects. Overall, the combination of J2H-1702 and nintedanib resulted in the greatest improvement of PF induced by irradiation [Fig. 7C].

## Discussion

The etiology of most chronic human diseases is complex, involving a mix of genetic and environmental influences that interact with each other over timespans ranging from hours to years. PF has diverse genetic and environmental causes, including long-term exposure to toxins, smoking, radiation therapy, medicines such as methotrexate and bleomycin, and certain inflammatory diseases. After the COVID‑19 pandemic, it became apparent that patients who survive the acute phase of COVID-19 pneumonia are at risk of developing deleterious consequences on lung function, most notably PF. Accordingly, the unmet medical need for PF treatment is expected to rapidly increase in the future. Because PF is caused by complex factors, drug discovery to overcome PF is challenging. The major causes of PF commonly involve excessive accumulation of ROS and inflammatory substances in lung tissue. Hence, compounds with antioxidant and anti-inflammatory actions are receiving attention as potential treatments for PF.

Oxidative stress and the production of extracellular matrix and inflammatory cytokines play crucial roles in the development of a fibrotic environment in multicellular spheroid models. Using multicellular hepatic spheroids, we previously determined that J2H-1702, a pharmacological inhibitor of 11βHSD1, relieves liver fibrosis by inhibiting pro-fibrotic gene expression and NF-κB signaling, which is triggered by pro-inflammatory cytokines such as TNF-α, IL-6, and IFN-γ in mouse models of liver fibrosis [18]. In the present study, we asked whether J2H-1702 might also be effective against PF, and we studied the mechanism by which J2H-1702 exerts its anti-fibrotic function using MCLSs containing fibroblasts and ECs to mimic fibrotic complexity in vitro. We were particularly intrigued by the previous finding that realistic reciprocal crosstalk between non-small cell lung cancer cells and HUVECs facilitates the development of compactness of MCLS architecture by modulating the EndMT process [13]. In the present study, we showed that J2H-1702 reversed the EndMT process to alleviate these architectural changes in MCLSs [Fig. 1].

PF is a chronic oxido-inflammatory disorder of the lungs. Because bleomycin-induced PF is associated with increases in ROS and TGF-β1, we mainly used bleomycin and TGF-β1 as triggers for EndMT. J2H-1702 suppressed EndMT induced by TGF-β1 or bleomycin in HUVECs and HCAECs by increasing HO-1 (encoded by *Hmox1*) expression [Fig. 2]. HO-1 is an inducible enzyme responsible for degrading free heme to iron and is primarily considered an antioxidant. The immunomodulatory and anti-inflammatory functions of HO-1 have been well established using various experimental animal models of inflammation [29–32]. Hence, targeting HO-1 expression and activation has attracted attention as a new therapeutic strategy to treat fibrotic diseases, including liver fibrosis, renal fibrosis, and PF.

Our results showed that pharmacological inhibitions and genetic manipulations of 11βHSD1 that promote activation of the Nrf2/HO-1 pathway not only protect against oxidative stress-induced cell damage in human vascular ECs [Figs. 3 and 4] but also drive the phenotypic shift to M2 macrophages [Fig. 5]. 11βHSD1 plays a key role in the regulation of hormones, metabolism, and inflammatory cytokines [33–35]. 11βHSD1 activity is markedly induced by pro-inflammatory cytokines such as IL-1β and TNF-α [36], and 11βHSD1 inhibition reduces inflammation in inflammatory diseases. Hence, 11βHSD1 inhibitors have been proposed as a novel strategy for the prevention and treatment of inflammatory disease. The selective 11βHSD1 inhibitor KR-66344 suppressed inflammation by inducing HO-1 expression [37]. BVT.2733, another specific 11βHSD1 inhibitor, ameliorated inflammatory responses, ROS production, and mitochondrial damage in LPS-stimulated THP-1 cells [33]. In the present study, we determined that 11βHSD1 inhibition by J2H-1702 reduced the production of pro-inflammatory cytokines, particularly IL-1β, and drove macrophage M2 polarization, which has anti-inflammatory effects.

To analyze the effect of 11βHSD1 inhibition on lung fibrosis in vivo, we employed two different mouse models of PF (bleomycin-induced idiopathic PF and RIPF) that efficiently induce severe damage and progressive fibrosis in the lungs [Figs. 6 and 7]. Although treatment with the 11βHSD1 inhibitor alone showed good therapeutic effects, co‑treatment with the 11βHSD1 inhibitor and nintedanib showed even better synergistic therapeutic effects on inflammation and fibrosis in both the bleomycin-induced idiopathic PF model and the RIPF model. Combination drug therapy is widely used across a range of diseases to enhance efficacy and improve outcomes, and there is a need to explore combination therapies to overcome the therapeutic limitations of current PF treatments. For example, clinical research on the safety and efficacy of combination therapy with nintedanib and pirfenidone is being conducted for patients with idiopathic PF [38, 39]. Our results show that J2H-1702 markedly improved the efficacy of nintedanib on lung fibrosis in vivo. This suggests that combined treatment with an 11βHSD1 inhibitor and nintedanib may be a promising approach to overcome lung fibrosis and treat EndMT-related disorders.

**Fig. 8:**
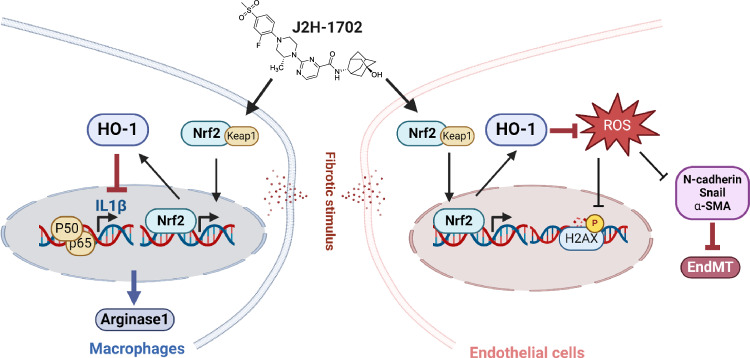
J2H-1702 controls EndMT and M2 macrophage polarization by elevating heme oxygenase-1. J2H-1702 controls EndMT and M2 macrophage polarization by elevating heme oxygenase-1 (HO-1). HO-1 regulates reactive oxygen species-induced DNA damage in endothelial cells and also regulates IL-1β and arginase 1 in macrophages.

In conclusion, our experiments using idiopathic PF and RIPF mouse models and morphometry analysis of MCLSs provide new indications for using the 11βHSD1 inhibitor J2H-1702 to treat lung fibrosis. J2H-1702 controls EndMT and macrophage polarization, facilitating robust therapeutic activity when used in combination with nintedanib to treat PF in mouse models. [Fig. 8]. Together, our data suggest that J2H-1702 can be considered a promising clinical candidate for the treatment of PF.

## Materials and methods

### Chemical agents

Bleomycin was purchased from Selleck Chemical (#S1214, Houston, TX, USA) for in vitro or Sigma (#B5507; Sigma-Aldrich, Louis, MO, USA) for in vivo experiments. TGF-β1 was purchased from Peprotech (#100-21C-10ug, NJ, USA). J2H-1702 was synthesized by J2H Biotech company. DMSO and Hydrogen peroxide (H_2_O_2_) 30% (w/w) solution were purchased from Sigma. Nintedanib was purchased from Selleck Chemical. PMA (Phorbol 12-myristate-13-acetate) was purchased from Sigma. Lipophilic tracer was purchased from Invitrogen (Waltham, MA, USA).

### Cell line and cell culture

A549 cells, NCI-H460 cells, and H1299 cells were obtained from the Korean Cell Line Bank. Human umbilical vein endothelial cells (HUVECs) and Human coronary artery endothelial cells (HCAECs) were obtained from Promocell. WI38 and THP-1 were purchased from the American Type Culture Collection (AATC; Manassas, VA, USA). The cells were maintained at 37 °C in a humidified atmosphere of 5% CO_2_. H460, H1299, and WI38 cells were cultured in Roswell Park Memorial Institute medium (RPMI 1640; Welgene, Korea) supplemented with 10% fetal bovine serum (FBS; Gibco, Grand Island, NY, USA), 1% penicillin-streptomycin (P/S; Gibco, Grand Island, NY, USA) (complete medium). A549 cells were cultured in Dulbecco’s Modified Eagle medium (DMEM; Welgene) supplemented with 10% FBS and 1% P/S. HUVECs and HCAECs were cultured in an endothelial cell growth medium supplemented with 0.5% P/S purchased from Promocell. THP-1 monocytes were differentiated into macrophages by 24 h incubation with 50 ng/ml PMA (Phorbol 12-myristate-13-acetate).

### Generation of multicellular lung spheroids (MCLSs)

To generate MCLSs, lung cells (A549, H460, H1299) were seeded with stromal cells (WI38 and HUVECs or HCAECs) at a total density of 6 × 10^3^ cells/well in 96-well round-bottomed ultra-low attachment microplates (Corning B.V. Life Sciences, Amsterdam, Netherlands). The cells were incubated for 3 days at 37 °C in a humidified atmosphere of 5% CO_2_ to generate MCLSs. J2H-1702 compounds were treated with MCLSs for 3 days.

### Immunocytochemistry and staining

Cells were fixed with 4% Paraformaldehyde (PFA, Biosesang, Gyeonggi-do, Korea) for 10 min, followed by incubation with primary antibodies in 5% BSA overnight at 4° C. After washing, the cells were incubated with a fluorescent-conjugated secondary antibody (Thermo Scientific). The nucleus was stained by Hoechst 33342 (Sigma-Aldrich, St. Louis, MO, USA). Reactive oxygen species (ROS) accumulation was detected by staining15, line 9) with CM-H_2_DCFDA in live cells. To visualize the spatial arrangement of the endothelial cells in MCLS, the HUVEC cell was stained using lipophilic tracers (DiD, fluorescence excitation (Ex) 644 nm/emission (Em) 665 nm). MCLS spheroids were fixed with 4% PFA for 30 min followed by incubation with CD31 primary antibody in 10% normal goat serum overnight at 4 °C. After washing, the MCLSs were incubated with a fluorescent-conjugated secondary antibody. The nucleus was stained by Hoechst 33342 (Sigma-Aldrich, St. Louis, MO, USA). All images were acquired using the Opera Phenix and analyzed by Harmony software.

### Cytokine array

THP-1 macrophage cells were rinsed with PBS and lysed using RIPA buffer supplemented with a proteinase inhibitor cocktail. Lysates were incubated in ice for 30 min. Microcentrigufe at 13,000 rpm for 10 min, and transfer the supernate into a clean tube. Protein lysates were used to run the proteome profiler human cytokine array (#ARY005B, R&D Systems, MN, USA). All procedures were performed according to the manufacturer’s instructions. Normalization against reference spots was performed.

### SDS-PAGE and Western blot

2D or 3D cells were lysed using radioimmunoprecipitation assay (RIPA) lysis and extraction buffer (Thermo Scientific), and measured protein concentration was with a BCA protein assay kit (Pierce, Wisconsin, USA). Calculated proteins were boiled with 5× sodium dodecyl sulfate-polyacrylamide gel electrophoresis (SDS-PAGE) loading buffer (Curebio, Korea) for 5 min, 100°C. Cell lysates were separated by 8–15% SDS-PAGE and transferred to a nitrocellulose (NC) membrane (Pall Corporation, Port Washington, NY, USA). Membranes were blocked with 5% skim milk in Tris-buffered saline/Tween 20 (TBS-T) buffer for 1 h, room temperature. After washing for three times in 10 min with TBS-T buffer, NC membranes were incubated with rabbit monoclonal anti-alpha Smooth Muscle Actin (α-SMA, E184), rabbit polyclonal anti-HSD11B1, rabbit polyclonal anti-N Cadherin, rabbit polyclonal anti-CD86, rabbit polyclonal anti-CD163, mouse monoclonal anti-Vimentin (RV202), mouse monoclonal anti-CD206, mouse monoclonal anti-HLA-DR, mouse monoclonal anti-Nrf2 (all from Abcam, Cambridge, UK), rabbit monoclonal anti-VE-cadherin, rabbit monoclonal anti-Snail, rabbit monoclonal anti-Epiregulin, rabbit monoclonal anti-iNOS, rabbit polyclonal anti-HO-1, mouse monoclonal anti-CD31, mouse monoclonal anti-Lamin A/C, mouse monoclonal anti-GAPDH (all from Cell Signaling Technology, Danvers, MA, USA), mouse monoclonal anti-SM22α (Transgelin) (Santa Cruz Biotechnology, Dallas, TX, USA), rabbit polyclonal anti-Arginase I (Novus Biologicals, Centennial, Colorado, USA), rabbit polyclonal anti-phospho-Histone H2AX (Merck Millipore, Burlington, MA, USA), and mouse monoclonal anti-β-actin (Sigma-Aldrich, St. Louis, MO, USA) for 16 h at 4 °C. After washing for three times in 10 min with TBS-T buffer, the membranes were incubated with horseradish peroxidase (HRP)-conjugated secondary antibody (Cell Signaling Technology, Danvers, MA, USA), and the specific bands were visualized by enhanced chemiluminescence (ECL; Thermo Scientific, Waltham, MA, USA). The full-length uncropped original western blots are described as supplemental material.

### Subcellular fractionation

A subcellular protein fractionation kit for cultured cells was purchased from Thermo Scientific. All procedures were performed according to the manufacturer’s instructions.

### RNA-seq analysis

The selection of DEGs was based on the RNAseq results using CLRNAseq^TM^ Software (http://www.chunlab.com/software_clrnaseq_download; Chunlab, Seoul, Korea). The data were normalized using a trimmed mean of *M*-value (TMM). Genes that were up- or down-regulated more than twofold were selected. Ensemble, FunRich, String DB, and Reactome were used as references. Functional pathway analysis was performed with protein-coding genes.

### Reverse transcription-polymerase chain reaction (RT-PCR) and quantitative real-time polymerase chain reaction (qReal-time PCR)

Total RNA was isolated from cells using the Rneasy mini kit (Qiagen) according to the manufacturer’s instructions. Reverse transcription-polymerase chain reaction was performed using iScript cDNA synthesis kit (BIO-RAD). The reaction mixtures were incubated at 25 °C for 5 min, and the transcription reaction was terminated by heating the mixture to 46 °C for 20 min. The reaction mixtures were inactivated at 95 °C for 1 min and then rapidly cooled on ice. For quantitative real-time PCR, the reaction mixture composed of SYBR Green (Applied Biosystems, Waltham, MA, USA) was used, and the reactions were performed using a ViiA 7 (Applied Biosystems). Primers were designed and purchased from Bioneer (Gyeonggi-do, Korea) or Cosmogenetech (Seoul, Korea). The primer sequences are described in Supplementary Table.

### Determination of HO-1, IL-1α, and IL-1β expression level

Heme Oxygenase 1 (HO-1) expression was measured by a Human Heme Oxygenase 1 simple step ELISA Kit (Abcam, Cambridge, UK). The Human IL-1α ELISA Kit and Human IL-1β ELISA Kit were purchased from Abcam. All procedures were performed according to the manufacturer’s instructions.

### Animal study—BLM (bleomycin-induced PF) model and RIPF (radiation-induced PF) model

#### BLM model

Six-week-old male mice (C57BL/6) were purchased from Orient Mice Co., Ltd. Bleomycin (1.6 U/kg) dissolved in 60 µL PBS was injected intratracheally into mice. Oral administration of 60 mg/kg nintedanib (5% DMSO + 30% PEG + 1% Tween 80), 30 mg/kg J2H-1702 (2.5% HP-β-CD), or a combination of nintedanib and J2H-1702 was initiated 14 days after bleomycin administration. The Drugs were orally administered daily for 2 weeks.

#### RIPF model

Radiation was administered using the X-RAD 320 (Precision X-ray, North Branford, CT, USA). The left main bronchi of 8-week-old C57BL/6 mice were irradiated with 90 Gy using a 4 mm diameter field. One hour before irradiation, 60 mg/kg nintedanib (5% DMSO + 30% PEG + 1% Tween 80), 30 mg/kg J2H-1702 (1.5% DMSO + 2.5% HP-β-CD), or a combination of nintedanib and J2H-1702 was administered orally. Subsequently, treatment administration was continued twice a day for 2 weeks.

#### Mice and ethical approval

All animal experiments are reported in accordance with the ARRIVE guidelines. Mice experiments were approved by the Institutional Animal Care and Use Committee (IACUC) of the Korea Institute of Radiological & Medical Sciences (Kirams 2021-0093, 2022-0099). All animal experimental data provided are representative of three independent experiments. All experiments were conducted using 6–8-week-old mice, and mice were maintained on a 12 h light-dark cycle in a standard environment (20 ± 1 °C room temperature, 50 ± 10% relative humidity) provided a standard diet and water *ad libitum*. All mice were anesthetized using a combination of anesthetics before being euthanized.

#### Immunohistochemistry staining

The tissues were immersed in a solution of 10% neutral buffered formalin for fixation, followed by embedding in paraffin, and then sectioned into tissue slices. Following deparaffinization, tissue slides were stained with H&E. The fibrosis grade was evaluated according to the Ashcroft score. Collagen deposition was evaluated through Masson trichrome staining using the Polyscience staining kit (#25088-100).

#### Microcone beam CT (micro-CBCT)

The micro-CBCT utilized the following settings: 40 kV and 3 mA with a small focal size, along with an Al filter that allowed adjustment of the tube voltage and x-ray tube settings. The system employed an amorphous silicon flat-panel detector manufactured by Perkin-Elmer in Wiesbaden, Germany, with a pixel size of 200 μm.

### Statistical analysis

Data were analyzed using GraphPad Prism 10 software (GraphPad Prism), and the results are presented as the mean ± standard deviation (SD) or mean ± standard error of the mean (SEM). Differences between groups were tested using the two-tailed, unpaired Student’s *t*-test. *P*-values below 0.05 are considered significant, and the following notation is used in the figures: *P < 0.05, **P < 0.01, and ***P < 0.001.

## Supplementary information

Primer sequences for qRT-PCR

RNA seq analysis

Original western blot data

## Data Availability

The datasets generated and/or analyzed during the current study are available from the corresponding author upon reasonable request.
