# Supplementary material for: Inhibition of 11β-hydroxysteroid dehydrogenase 1 alleviates pulmonary fibrosis through inhibition of endothelial-to-mesenchymal transition and M2 macrophage polarization by upregulating heme oxygenase-1
Source: Cell Death Dis. 2025 Mar 21;16(1):196. doi: 10.1038/s41419-025-07522-2 (PMC11928689; doi:10.1038/s41419-025-07522-2)
Supplement: Supplementary file 1 — Primer sequences for qRT-PCR [file 41419_2025_7522_MOESM1_ESM.docx]

**Supplementary Table. Primer sequences for qRT-PCR**

| **GENE** | **SEQUENCE (5' --> 3')** | | |
| --- | --- | --- | --- |
| EREG | forward |  | GTG ATT CCA TCA TGT ATC CCA GG |
|  | reverse |  | GCC ATT CAT GTC AGA GCT ACA CT |
| INHBE | forward |  | ATC TTC CGA TGG GGA CCA AG |
|  | reverse |  | AGA GTT AAG GTA TGC CAG CCC |
| FGFR2 | forward |  | AGC ACC ATA CTG GAC CAA CAC |
|  | reverse |  | GGC AGC GAA ACT TGA CAG TG |
| IL-1α | forward |  | TGG TAG TAG CAA CCA ACG GGA |
|  | reverse |  | ACT TTG ATT GAG GGC GTC ATT C |
| CMPK2 | forward |  | GTA CCT CCT TTA TTC CTG AAG CC |
|  | reverse |  | ATG GCA ACA ACC TGG AAC TTT |
| CHMP4C | forward |  | TGG TCC GAC TTC GGG AGA C |
|  | reverse |  | GCC AGG GCG ATT TCT CTC TG |
| RAB39B | forward |  | GAG GCC ATC TGG CTG TAC C |
|  | reverse |  | ACG GTG GGG TCA GAA ACC T |
| MKX | forward |  | CTC GCA GAT GAC GCT AGT GC |
|  | reverse |  | TGG CTG TCG AAC GGT ATT CTT |
| OASL | forward |  | CTG ATG CAG GAA CTG TAT AGC AC |
|  | reverse |  | CAC AGC GTC TAG CAC CTC TT |
| HO-1 | forward |  | AAG ACT GCG TTC CTG CTC AAC |
|  | reverse |  | AAA GCC CTA CAG CAA CTG TCG |
| Arginase 1 | forward |  | GTG GAA ACT TGC ATG GAC AAC |
|  | reverse |  | AAT CCT GGC ACA TCG GGA ATC |
| IL-1β | forward |  | ATG GCA TGG AGA ATG AAC ACC G |
|  | reverse |  | TGA TGT CTT CCG ACC TGC TGG T |
| β-actin | forward |  | TCG TGC GTG ACA TTA AGG AG |
|  | reverse |  | AGC ACT GTG TTG GCG TAC AG |
