# Supplementary material for: Inhibition of 11β-hydroxysteroid dehydrogenase 1 alleviates pulmonary fibrosis through inhibition of endothelial-to-mesenchymal transition and M2 macrophage polarization by upregulating heme oxygenase-1
Source: Cell Death Dis. 2025 Mar 21;16(1):196. doi: 10.1038/s41419-025-07522-2 (PMC11928689; doi:10.1038/s41419-025-07522-2)

Fig 1C.

Full and uncropped western blot for Figure 1C

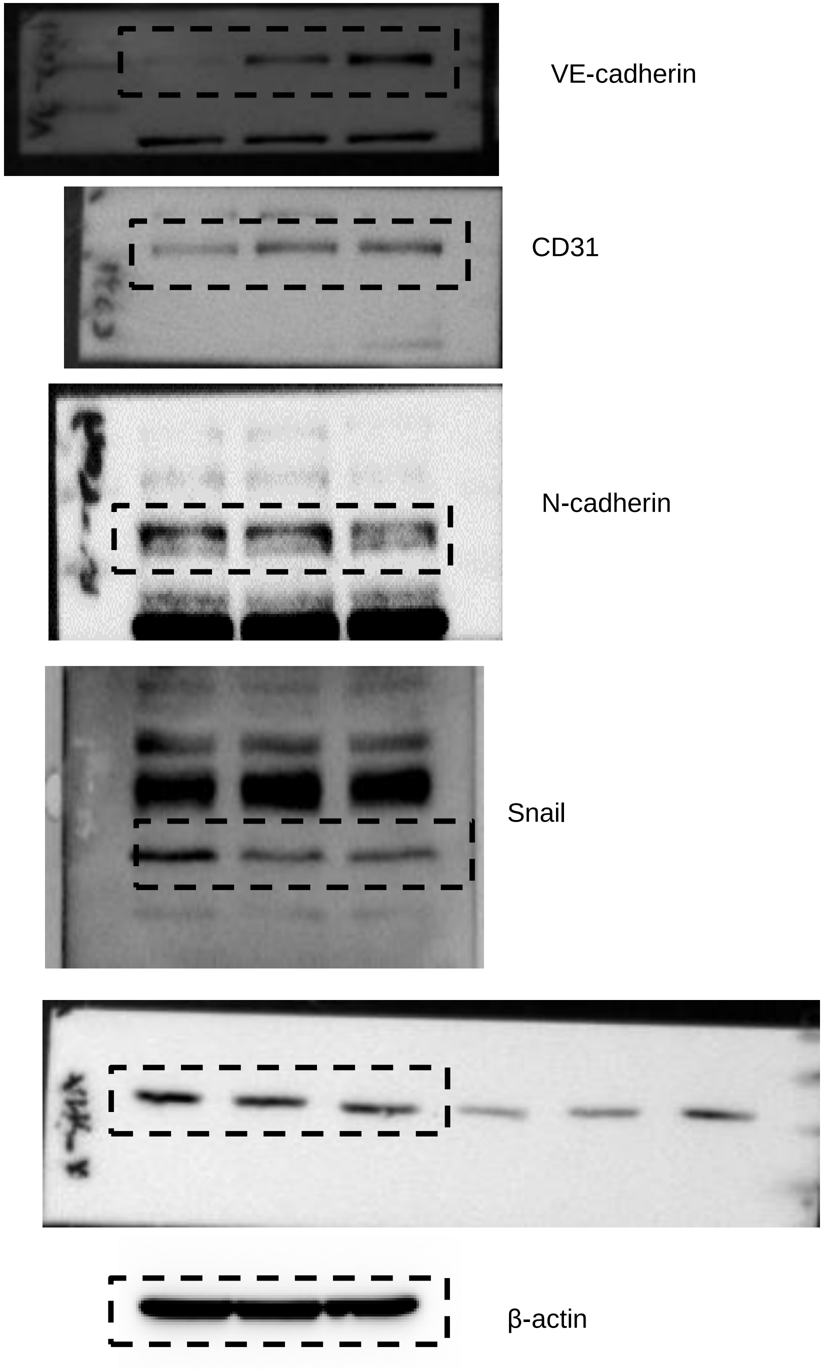

VE-cadherin

CD31

N-cadherin

Snail

β-actin

Full and uncropped western blot for Figure 1E, F, H, I

Fig 1E.

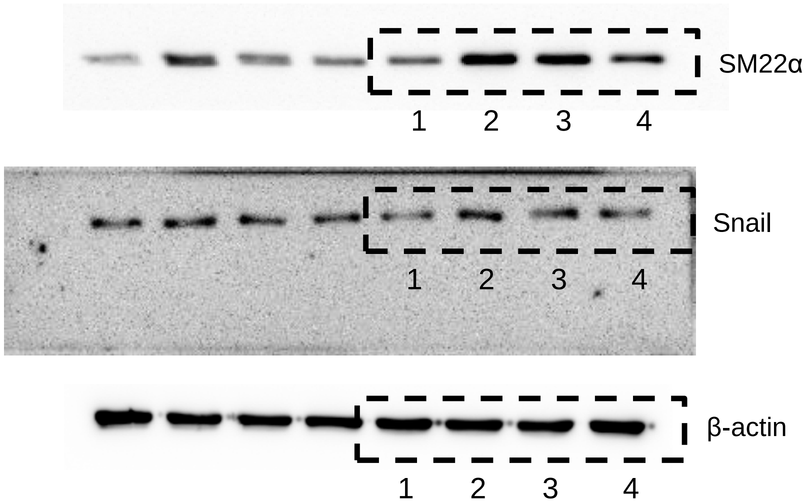

Fig 1F.

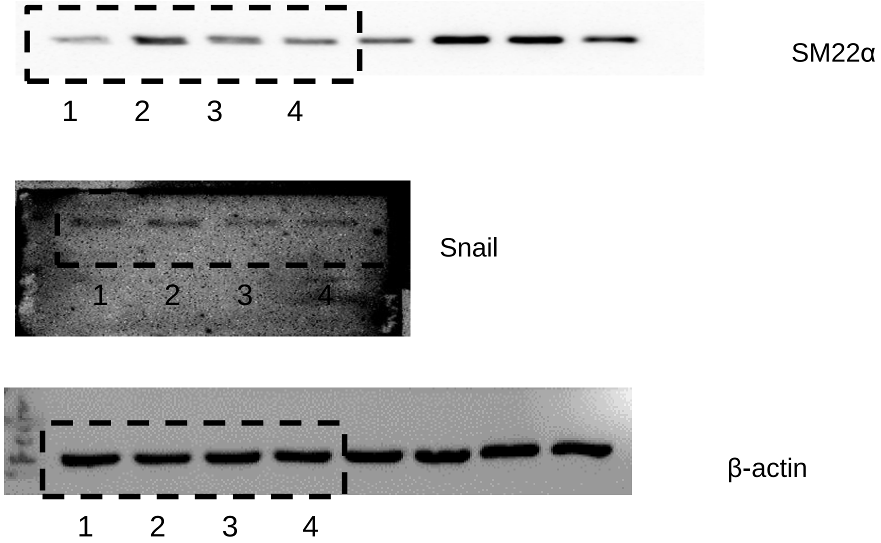

Fig 1H.

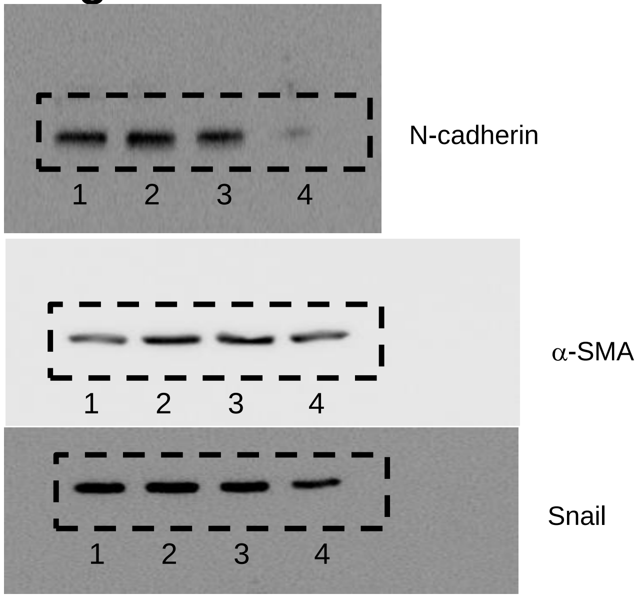

Fig 1I.

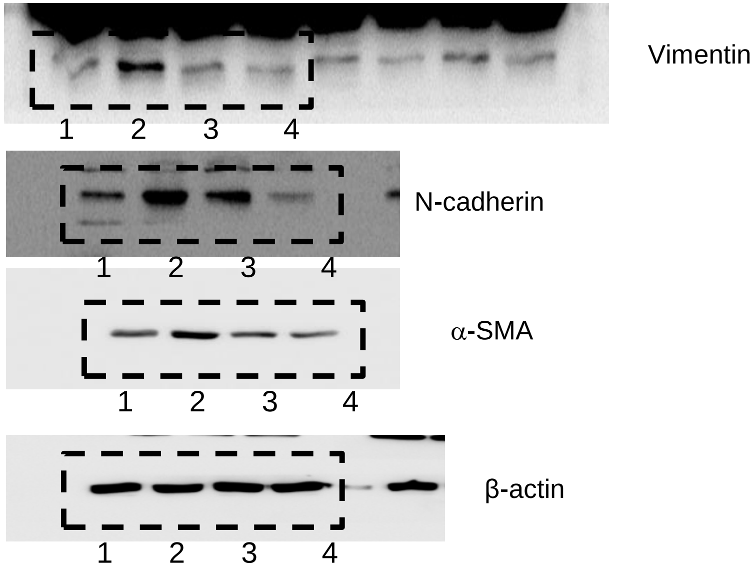

Full and uncropped western blot for Figure 2B

Fig 2B.

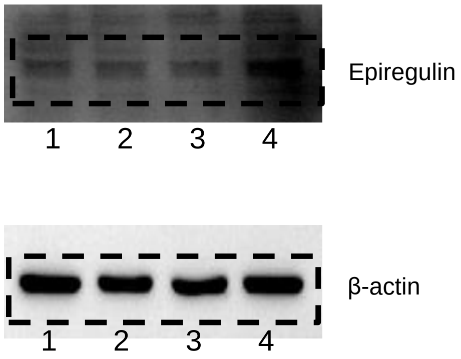

Full and uncropped western blot for Figure 3C, D

Fig 3C.

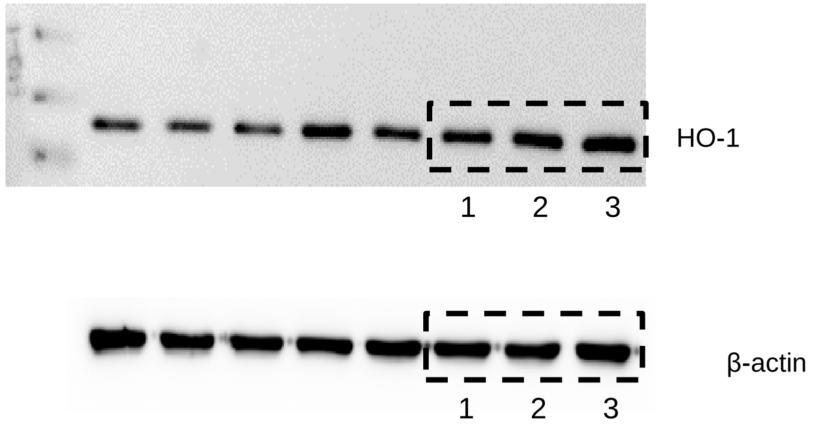

Fig 3D.

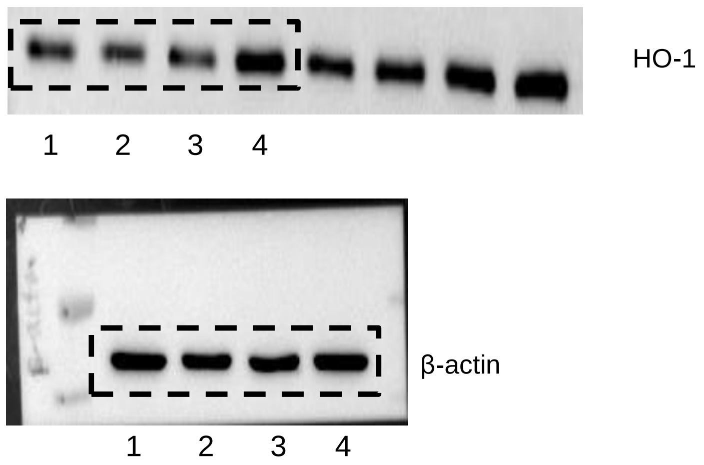

Full and uncropped western blot for Figure 3F, H

Fig 3F.

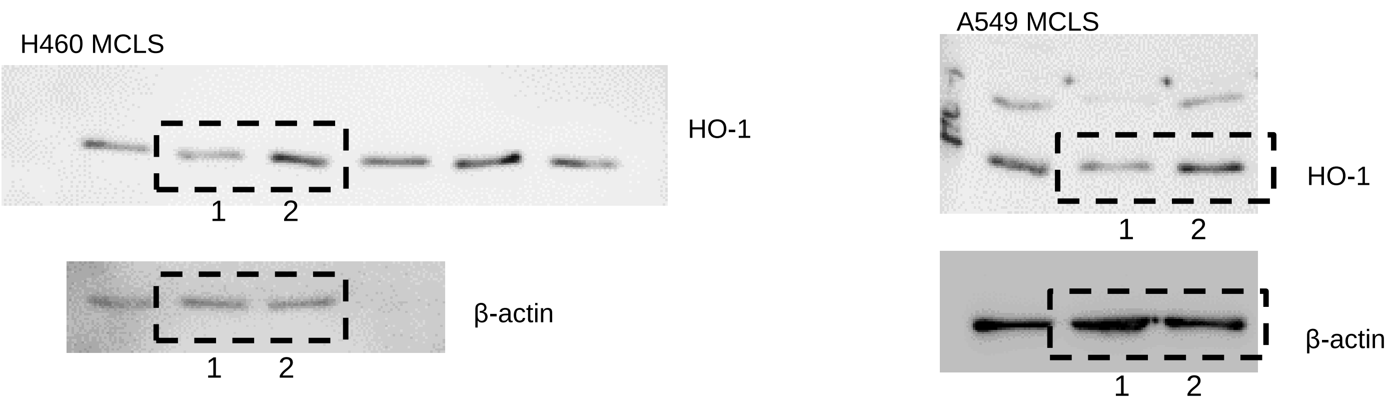

Fig 3H.

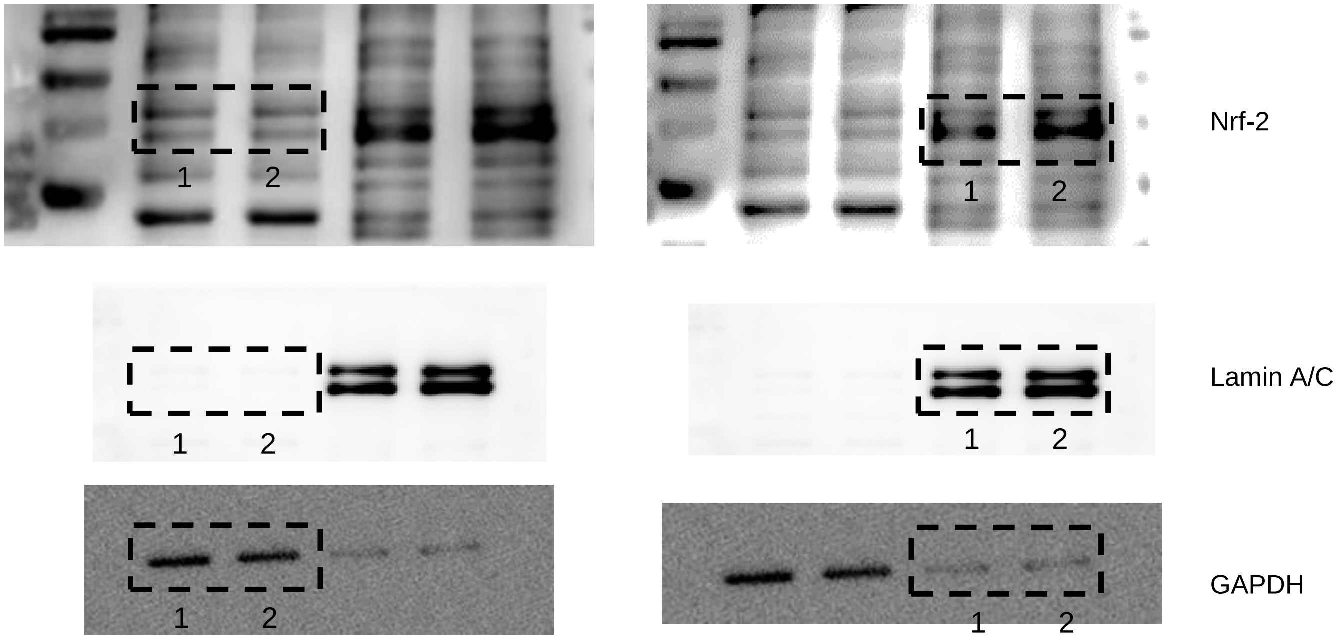

Full and uncropped western blot for Figure 3I, J

Fig 3I.

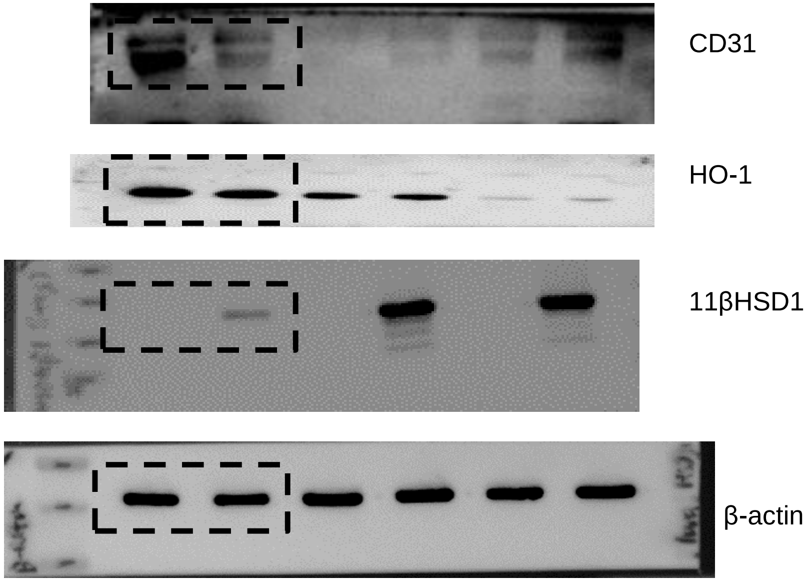

Fig 3J.

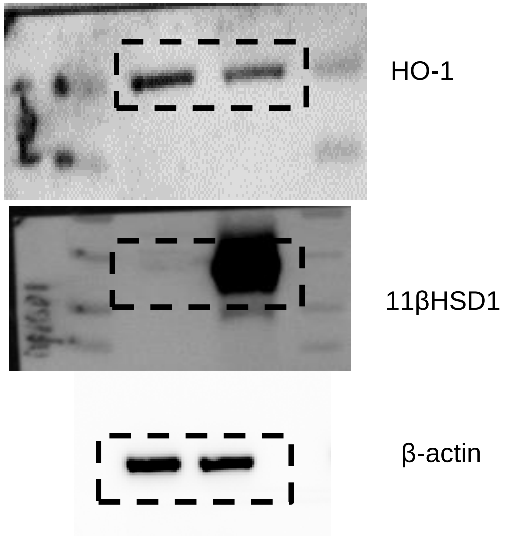

Full and uncropped western blot for Figure 4C, D

Fig 4C.

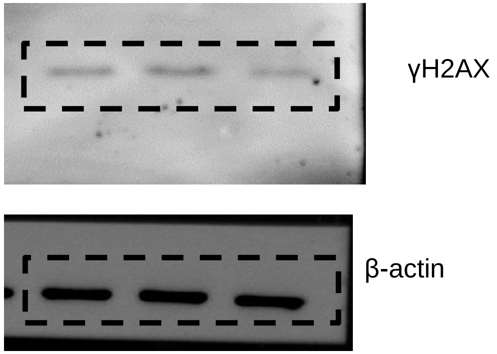

Fig 4D.

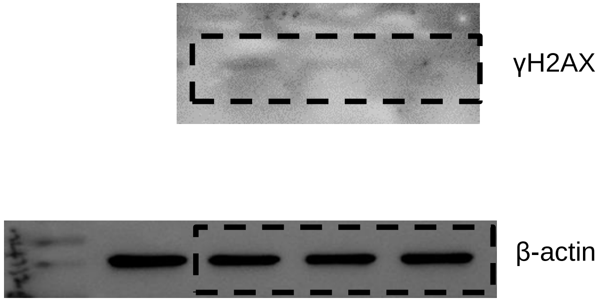

Full and uncropped western blot for Figure 5A, D

Fig 5A.

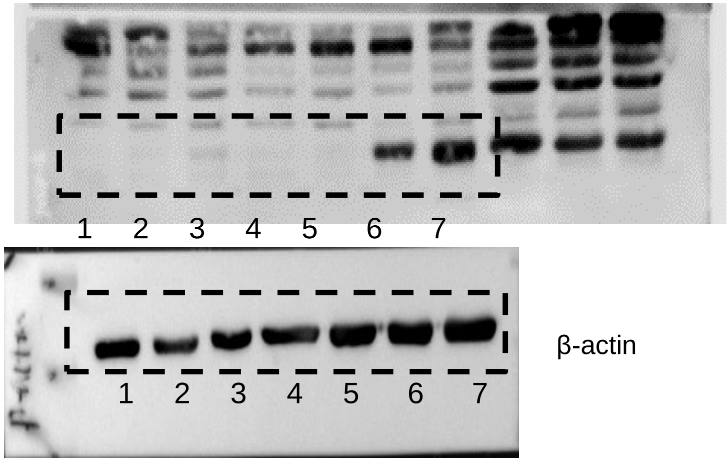

Fig 5D.

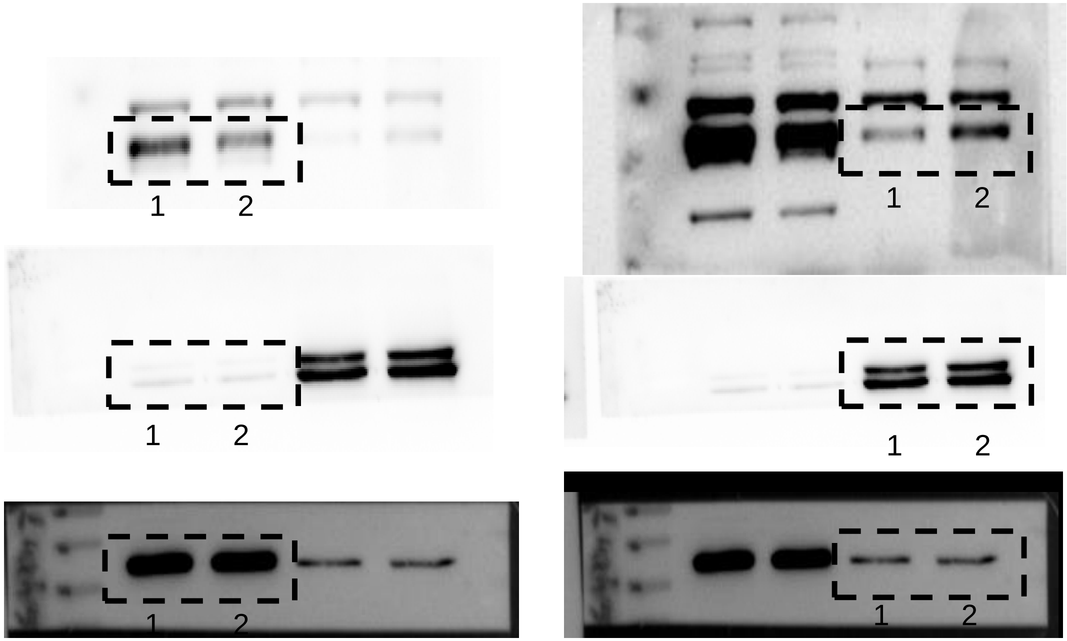

Full and uncropped western blot for Figure 5E, F

Fig 5E.

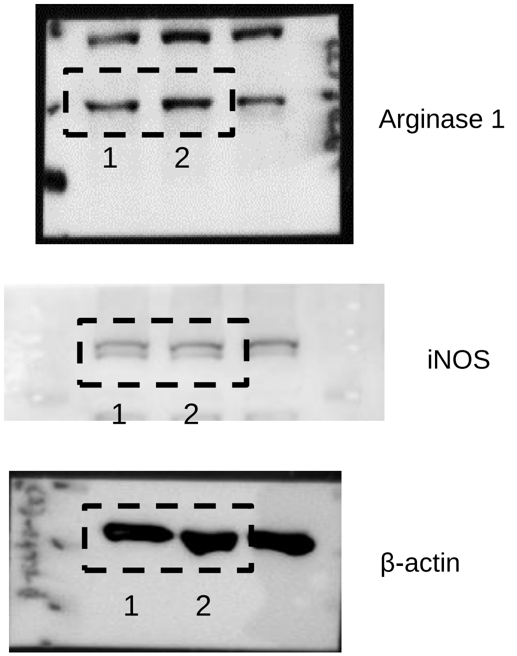

Fig 5F.

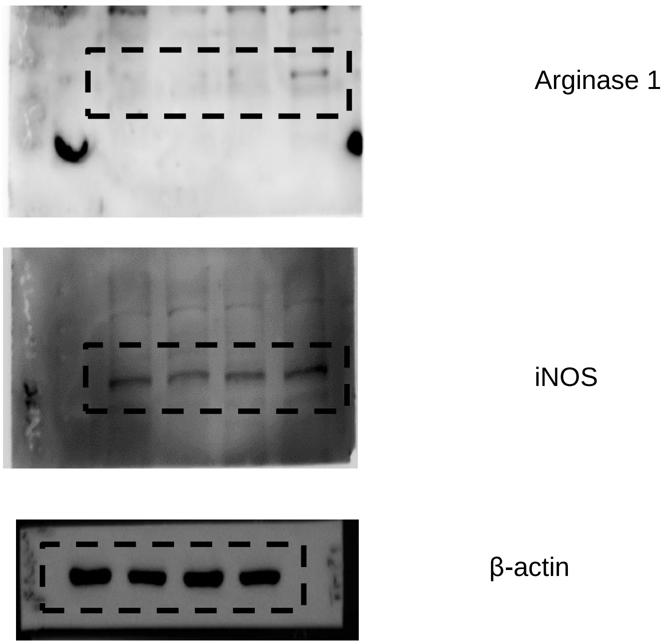

Supplement: Supplementary file 3 — Original western blot data [file 41419_2025_7522_MOESM3_ESM.pdf]
